# Experiences of individually tailored physical exercise in COVID-19-induced postural orthostatic tachycardia syndrome: A qualitative content analysis

**DOI:** 10.1371/journal.pone.0360748

**Published:** 2026-11-04

**Authors:** Annie Svensson, Pernilla Sönnerfors, Linda Holmström, Malin Nygren-Bonnier, Anna Svensson-Raskh

**Affiliations:** 1 Division of Physiotherapy, Department of Neurobiology, Care Sciences and Society, Karolinska Institutet, Stockholm, Sweden; 2 Medical Unit Allied Health Professional, Women’s Health and Allied Health Professional Theme, Karolinska University Hospital, Stockholm, Sweden; 3 Department of Medicine Solna, Karolinska Institutet, Stockholm, Sweden; 4 Department of Clinical Neuroscience, Karolinska Institutet, Stockholm, Sweden; 5 Department of Women’s and Children’s Health, Karolinska Institutet, Stockholm, Sweden; Somaiya Vidyavihar University K J Somaiya College of Engineering, INDIA

## Abstract

**Background:**

Postural Orthostatic Tachycardia Syndrome (POTS) is a phenotype of post-COVID-19 Condition (PCC), characterised by orthostatic intolerance and a range of debilitating symptoms. Exercise is a frequently implemented intervention in the treatment of POTS and PCC. However, patients’ experiences with exercise as a therapeutic modality remain largely unexplored. This study aimed to explore the experiences of individuals diagnosed with PCC and POTS who underwent an individually tailored physical exercise intervention.

**Methods:**

A qualitative study design with an inductive approach was applied. Twenty-three participants who completed a feasibility study, involving semi-supervised tailored exercise, were recruited for individual interviews. Data were collected using a semi-structured interview guide and analysed through qualitative content analysis.

**Results:**

Five main categories emerged, underscoring the importance of individualised design of exercise programs, flexible modalities, and continuous support from physiotherapists with expertise in PCC and POTS. Participants experienced improvements in physical, mental, and social functioning, although setbacks and symptom fluctuation were common. Strategies for energy management, alongside informal and formal support, were identified as facilitators and reduced barriers to exercise as a treatment. Psychological dimensions, including hope, motivation, goal setting, emotional adaptation, acceptance, and coping strategies, were central to participants’ experiences. The combination of the intervention structure, therapeutic support, and effective strategies was perceived as contributing to the intervention’s effectiveness, and many participants viewed exercise as a key pathway to recovery.

**Conclusions:**

This study highlights the need for a flexible and individualised rehabilitation approach in COVID-19-induced POTS. Moreover, exercise should be closely monitored by a physiotherapist with condition-specific expertise, who not only adapts the exercise to symptom fluctuations but also addresses diverse psychosocial needs and individual resources. The participants’ experiences indicate that a holistic, multidisciplinary, and person-centred rehabilitation model may support recovery and contribute to improved long-term wellbeing in individuals with PCC and POTS.

## Introduction

Already during the first wave of the COVID-19 pandemic, reports emerged from patients, describing persistent and debilitating symptoms following the acute infection, and the World Health Organisation (WHO) defined Post-COVID-19 Condition (PCC) in September 2020 [1]. The prevalence of PCC is estimated to be 1.8% − 6.7% [2,3] and seems to be sustained over time [4]. In 2021, over 29,000 cases of PCC were reported in Sweden [5], however, the current number of cases remains unknown. The demographic profile of PCC in Sweden is similar to global reports, with a majority of affected individuals being females aged 18–69 years [5]. Certain characteristics of the acute COVID-19 illness and its treatment, as well as individual attributes such as being unvaccinated, female, aged 35–50 years, having pre-existing comorbidities, or a higher body mass index (BMI), increase the risk of developing PCC [2,3,6]. PCC can also co-exist with or cause other symptoms, and several studies have highlighted dysautonomia and postural orthostatic tachycardia syndrome (POTS) as a specific phenotype of PCC (PCC-POTS) [7–12]. The prevalence of PCC-POTS is reported to be approximately 30% [9,11]. The estimated proportion of individuals affected by POTS in Sweden aligns with international reports [13], and in 2021, 15% of individuals with PCC also had a POTS diagnosis [5]. POTS is characterised by orthostatic intolerance and associated symptoms such as nausea, dizziness, palpitations, fatigue, sleep disturbances, muscle weakness, tremors, headache, and cognitive problems [14–18]. The symptoms of POTS and PCC negatively affect health-related quality of life, physical and mental health, work capacity, activities in daily life and decrease social interactions [19–28].

Currently, there is no evidence-based treatment aiming to cure PCC [3,6], but available approaches focus on alleviating the symptoms of PCC [29,30]. Most interventions for PCC are rehabilitative or non-pharmacological [29,31], and emerging evidence suggests that physical exercise may be an effective treatment [32,33]. The treatment of POTS includes both pharmacological and non-pharmacological approaches [14,16–18,34–36], and physical exercise is a method that can improve symptoms, physical fitness, and quality of life [35]. However, recent publications [37,38] highlight the need for individualised treatment. Recommendations for POTS are often applied to PCC-POTS [12,15,39], although the evidence of effectiveness in this subgroup remains limited [35,39]. Moreover, although a few studies have explored patients’ experiences of rehabilitation [40–43] or exercise [44–48], important questions remain, particularly regarding experiences of exercise-based interventions among individuals with POTS or PCC-POTS. Importantly, participants in our previous feasibility study [49] provided valuable insights into how such interventions were experienced, underscoring the necessity for a more comprehensive qualitative investigation. Therefore, the overall aim of this study was to explore and describe how people with PCC-POTS experience individually tailored physical exercise as a treatment.

## Methods

### Design

The study was designed as a qualitative study with an inductive approach, registered at Clinical Trials (NCT05094622), and reported according to the criteria for reporting qualitative research (COREQ) [50]. The study was approved by the Swedish Ethical Review Authority (2020–02149, including amendments) and conducted in accordance with the Declaration of Helsinki and Good Clinical Practice. All participants provided written informed consent prior to participation.

### Setting and context

During 2022, a feasibility study was conducted at Karolinska University Hospital and Karolinska Institutet in Sweden. The study aimed to evaluate the feasibility of individually tailored exercise for patients with PCC-POTS [49]. The trial assessed the feasibility of a twelve-week intervention consisting of individually tailored endurance and strength exercises. The intervention was delivered within clinical care by physiotherapists at the Karolinska University Hospital or in the primary care setting in Stockholm. The participants had regular contact with the physiotherapist during the intervention to adjust the treatment recommendations over time [49].

### Participants

A criterion-based sampling was used, and all participants who participated in the feasibility study by Svensson et al [49], had received the intervention, and remained in the study at the time of recruitment for the interview were considered eligible, i.e., a maximum of 25 possible participants.. Two eligible participants could not be reached for inclusion, but the remaining eligible participants all agreed to participate in the interviews, resulting in 23 participants included. The demographics are presented in Table 1.

**Table 1:** Demographics of participants, n = 23.

Female, *n*	19		
Age, *median (Min-Max)*	44 (24-57)		
Higher education, *n*	18		
Occupation before COVID-19 illness, *n*	Healthcare		5
	Education and pedagogy		6
	Legal and administrative occupations		4
	Communication and culture		2
	Service occupation		3
	Technic and IT		1
	Student		2
Employment status, *n*	Working	Full-time	1
		Partial	5
	Sick leave	Full-time	13
		Partial	4
	Studying		4
Marital status, *n*	Married or partner		8
	Single		13
	Missing		2
Parental status*, *n*	7		
COVID-19 History	Months since the initial COVID-19 infection, *mean (SD)*		20.7 (5.0)
	Months since POTS diagnosis, *mean (SD)*		3.1 (2.2)
	Hospitalised at the acute infection, *n*		2
POTS Treatment, *n*	Compression garments		15
	Drugs targeting cardiovascular functions		22
Previous physiotherapy or exercise treatment aimed at treating PCC, *n*	16		

* Caring for children under age 18, SD = Standard Deviation, POTS = Postural Orthostatic Tachycardia Syndrome, PCC = Post-COVID-19 Condition,

### Data collection

Individual interviews were conducted between March 2022 and May 2023, approximately one month after the participants ended the feasibility study. Demographic data were collected through medical records, study protocol, or from participants’ narratives during the interviews. The participants chose whether the interview would be conducted by telephone, video, or face-to-face at Karolinska University Hospital. All participants chose telephone or video.

Two female interviewers conducted nine and 14 interviews, respectively. The interviewers had different professional backgrounds; one had a master’s degree and worked as a licensed health and medical care social worker, with a total of 14 years of professional experience (E.F.), and the other was a specialist physiotherapist with a doctoral degree who had 25 years of professional experience (P.S.). Both interviewers had experience from interviewing participants in other research projects, but none of the interviewers was involved in the previous study [49] or had any experience with the intervention or POTS diagnosis; however, one of the interviewers (E.F.) had some personal experiences with PCC.

The interviews were carried out individually and lasted between 38–98 minutes (median 63 minutes). A semi-structured interview guide, focusing on participants’ experiences of participating in the exercise intervention, was used (S1 Appendix). The interview guide aimed to cover experiences of performing exercise, effects after performing the intervention, and experiences of the various settings in the exercise intervention. After the first and second interviews, the quality of the interview guide was reviewed by interviewer E.F. and researcher A.S-R without any need for adjustments. All interviews were audio recorded, and participants could request the recorder to be turned off at any time during the interview. The interviews were transcribed verbatim by the research team or by a professional company. Participant checking was done if clarifications were deemed necessary or upon request from the participant.

### Data analysis

The analysis was performed according to the method of qualitative content analysis described by Elo and Kyngäs [51], and the software OpenCode 4 [52] was used to manage the data in the analysis. The analysis was prepared independently by two authors (A.S and A.S-R) by repeated readings of the transcripts and identifying units of analysis, as shown in Table 2. The two authors were both female specialist physiotherapists with 14 and 25 years of experience in cardiovascular and/or respiratory diseases, respectively, and both were involved in the feasibility trial [49] by conducting treatment (A.S) and data collection (A.S and A.S-R). Manifest open coding was performed, and a codebook was constructed through several joint discussions between the two authors until agreement was achieved; examples of the codebook are shown in Table 3. The other authors (L.H, M.N-B, and P.S) each read a selection of interviews and were then introduced to the codebook and were able to make comments, improvements, and/or validate the coding and the codebook. The final version of the codebook had a few changes made according to suggestions from L.H, P.S, and M.N-B, and was then used for the organisation phase of the analysis. Authors L.H. and M.N-B (both female physiotherapists and associate professors) were involved in the feasibility trial by designing and analysing, but had no relation to the participants in the trials.

The first author (A.S) made a draft of the organisation of the codes into groups of subcategories, generic, and main categories. The grouping and categorisation were then improved by analysing and refining the data in collaboration with all authors. The collaborative discussions and analysis of the subcategories and main categories were iterative, and the analysis was considered complete when a consensus on the coding organisation and results was reached. Quotes were then selected to describe and clarify what emerged within the categories.

**Table 2:** Example of selection of the unit of analysis and further organisation.

Unit of analysis	Code	Sub/Generic Category	Main Category
**“**And the best thing was, like, yeah, I actually got exercises that were tailored just for me.”	Individualised progression	Progression	Content and Structure of the Intervention

**Table 3:** Example of the code book used in the analysis, including codes and descriptive sentences.

Code	Description
Flexibility	Descriptions of the study design’s flexibility regarding where and when training could be performed
Changed Routines	Descriptions of how routines around physiotherapy follow-ups, and what changes in these routines meant for participants
Preventing Deterioration	Reflections on how participants believed their condition would have worsened without training
Expectations	Participants’ accounts of their expectations regarding the training before the intervention began
Training Frequency	Descriptions of how often training was performed and what effects this had
Frequency of Follow-ups with PT	Descriptions of how often follow-ups with the physiotherapist occurred, and reflections on what frequency was considered valuable

## Result

The analysis resulted in five main categories: “Content and structure of the intervention”, “The role of a physiotherapist”, “Impact of the tailored exercise”, “Prerequisites and Circumstances” and “Balancing hope and Expectations“. Each main category has generic categories, and some also have sub-categories. Each main category is elaborated upon individually, including the generic and sub-categories within each main category. The categorisation is presented in Fig 1.

**Fig 1:**
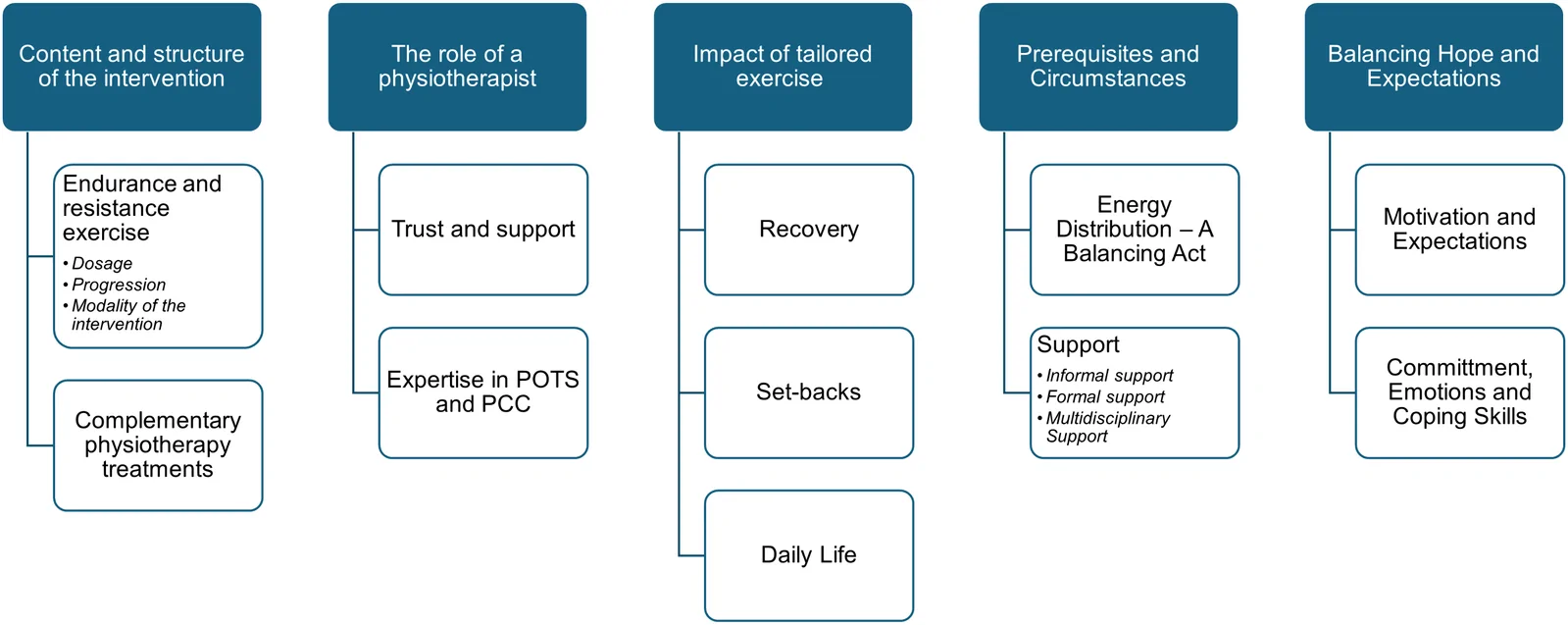
Illustration of the results with the five main categories: “Content and structure of the intervention”, “The role of a physiotherapist”, “Impact of tailored exercise”, “Prerequisites and circumstances”, and “Balancing hope and expectations”, with corresponding generic and sub-categories identified in the analysis.

### Content and structure of the intervention

This main category consists of two generic categories, “Endurance and Resistance Exercise” and “Complementary Physiotherapy Treatments”. In the category “Endurance and resistance exercise”, there are three sub-categories: “Dosage”, “Progression”, and ”Modality of the intervention”. These were all identified as very important for participants’ perception of the intervention and whether the intervention was experienced as feasible.

#### Endurance and resistance exercise.

A main finding of this category was the importance of introducing endurance exercises in the supine position and the use of an indoor pedal trainer, since this was the only way that many participants could exercise without orthostatic symptoms being triggered. The participants highlighted positive aspects of the design and choice of exercises, where simple or basic exercises were highly valued to facilitate the implementation of the exercise. The importance of being flexible in types of prescribed exercises and of continuously making individually tailored adaptations when needed, was emphasised by participants.

Participants reported difficulties engaging in both resistance and endurance training, with no clear preference on which modality was more challenging emerging from the material. Some experienced symptom exacerbation primarily from endurance activities, while others were affected by resistance exercises. The exacerbations seemed to occur despite individualised adaptations, including exercises performed in a supine position. Nevertheless, the importance of maintaining strength training was emphasised by the participants, as they perceived having muscle weakness related to PCC and POTS.

*“I have had strength exercises as well. I’m a bit surprised myself, I must say, because I thought it was the cardio that would be the hardest, and that might trigger symptoms or maybe deterioration too. But for me, it’s been the opposite, it’s actually been the strength training that has been the most difficult.”*

*-Participant A*

##### Dosage.

Participants described individually tailored interval exercise, both endurance and resistance, as beneficial, especially when structured into sets or time-based intervals. According to the participants, a key challenge was finding the right dosage of intensity and frequency and still preventing overexertion and symptom exacerbation. Structured training plans with scheduled rest days were generally appreciated, although some individuals reported requiring more frequent recovery periods than prescribed to maintain their well-being. Another way to avoid overexertion was to perform low-intensity exercise, though it was sometimes perceived as ineffective and trivial.

*“I felt that when I started, it was very, very low intensity — it barely felt like I wasn’t doing anything during the first two or three weeks. But then it started to increase, and I began to feel like, okay, now it’s getting a bit tougher. Now I can actually feel that I’m exercising, or … my legs felt like they were drained of blood and filled with a thousand ants all over…”*

*-Participant B*

These perceptions regarding low intensity were often influenced by participants’ prior activity levels and expectations, as previously effortless activities were now experienced as strenuous. Most participants used the Borg Rating of Perceived Exertion (RPE) scale to navigate the intensity, and while some found it helpful for self-regulation, others expressed frustration over the lack of objective physiological markers, such as heart rate, to quantify their exertion.

##### Progression.

A key factor for enabling progression was the gradual introduction of low-intensity exercise. The participants viewed this approach as a way to avoid overexertion and allowed for sustainable improvements over time. Certain weeks or periods of the program could be experienced as more challenging, and participants described that the flexibility in the type of exercises and dosage enabled them to continue exercising on an overall level, without symptom deterioration.

Participants described that progress was often facilitated by maintaining a stable training level over time rather than continuously increasing intensity. This plateau strategy, combined with very gradual increases in dosage, was described to reduce stress and allow physiological adaptation.

##### Modality of the intervention.

An important aspect of the design was the context in which the training was performed. Group-based training was perceived as particularly valuable, offering not only physical benefits but also social and psychological support through interaction with others experiencing similar symptoms. However, those who primarily conducted the intervention at home highlighted that even though they would have appreciated the social benefits of a group setting, it was not an option for them. This since fatigue, transportation demands, and the combined physical and social effort being barriers. Instead, they appreciated the flexibility to do the exercise in their home setting, since they would not have been able to exercise at all if they needed to attend a clinic.

#### Complementary physiotherapy treatments.

Since the intervention was individually tailored, other types of physiotherapy treatment were available to the participants and were prescribed by the physiotherapist according to the participants’ individual needs. The benefits of and occasional need for other types of physiotherapy interventions were identified by some participants. Some described having additional treatments such as breathing exercises, flexibility exercises or a combination of adding cognitive tasks into the physical exercise and described positive experiences from this. Other participants identify the lack of the same, where they experienced that only some of their symptoms were targeted by the exercise intervention, but they felt the need for complementary exercises, for example, treatments targeting breathing difficulties or promoting relaxation.

#### The role of a physiotherapist

Trust and validation in the relationship with the physiotherapists were central to participants’ engagement and sense of safety during rehabilitation. By offering support, knowledge and continuity in care, it became an essential part of the experiences of the intervention.

#### Trust and support.

The participants expressed strong appreciation for the physiotherapists, describing them as attentive, supportive and dedicated. Feeling seen and validated was essential for psychological well-being and sustained engagement in the rehabilitation process. Encouragement and motivation from the physiotherapist were particularly valuable.

Continuity of care emerged as a key facilitator for adherence to the exercise intervention. The continuity also fostered trust and active engagement in the rehabilitation and contributed to a sense of security and belonging among the participants. To have regular contact with the same physiotherapist/s enabled individualised goal setting and adjustments. The continuity and support were described as especially important during setbacks where participants needed to slow down or pause progression. It was valuable when the physiotherapist normalised setbacks and explained them and thereby reduced anxiety and guilt for the participants.

*“It was like having someone constantly pulling you back and saying, ‘No, you’re not allowed to push yourself more than this, you’re not allowed to push yourself more than this.’ That’s really important, because otherwise I do too much — I get too impatient and want this to work.”*

*-Participant B*

Despite the overall positive experience regarding support from physiotherapists, some participants reported occasional shortcomings, such as reduced attentiveness during busy periods or insufficient support when the rehabilitation progress stalled. Some participants also felt pressured by the regular follow-ups and found it difficult to express setbacks. They describe themselves as dependent on the physiotherapists and affected their sense of support and ability to maintain training.

*“What I found difficult was… I often had questions, and it was really nice to be able to talk them through. But the only thing I found hard was when I had setbacks and didn’t quite dare to increase the intensity, and then I felt it was a bit tough sometimes — like I wanted to say, ‘Yes, it’s going well, let’s increase,’ or something like that.”*

*-Participant C*

#### Expertise in POTS and PCC.

Participants express strong appreciation for physiotherapists with specific expertise in POTS and a holistic understanding of the condition and the patient’s situation. Being treated by physiotherapists who demonstrated knowledge of the syndrome’s complexity, including symptoms, underlying mechanisms, and comorbidities, was associated with increased safety and treatment efficacy by the participants. Participants who had previously experienced inadequate care from physiotherapists or exercise interventions, attributed this to the lack of condition-specific expertise and raised concerns about the risk of inappropriate or even harmful treatments leading to symptom deterioration.

*“… when you meet doctors or physiotherapists and you notice that they don’t know anything about this, then it becomes really… it’s hard to be a patient in that situation, because you don’t trust them. You must be careful and take what they say with a grain of salt. So, when I got to come to [name of the clinic delivering tailored exercise intervention] and meet people who had met others like me, it made a big difference — it meant a lot.”*

*-Participant D*

Clear, yet nuanced information from the physiotherapist, about expected progress and potential setbacks, was appreciated by participants. Somewhat insufficient or limited communication was also reported and was primarily experienced during periods of symptom deterioration or when alternative exercise modalities were requested. Participants expressed a need for written information from the physiotherapist, both concerning the intervention itself and the weekly prescriptions. They also emphasise that the information should preferably be tailored to individual needs. To promote autonomy, support communication and facilitate the unsupervised sessions, participants proposed enhancing the use of written materials or recorded content. The need for improved information was considered particularly evident among individuals experiencing cognitive impairments.

#### Impact of tailored exercise

This category explains the participants’ experiences of improvements and gradual recovery that the individually tailored exercise was associated with, but also their experiences of continuous and unpredictable setbacks during their illness and the period of intervention.

#### Recovery.

Individually tailored exercise was associated with a range of positive outcomes and improved recovery, including improvements in physical function, fatigue, mental health, and cognitive performance. Regarding bodily functions, participants reported improvements such as reduced dizziness, increased muscle strength and endurance. Examples of improved cognitive capacity were better managing cognitively demanding tasks such as reading, studying, or returning to work. Psychosocial benefits were also noted, including reduced mental fatigue, improved mood, and greater engagement in family and social life. Many described the outcome as a sense of returning to normality, though slow and gradual. Exercise was perceived as meaningful, which highly contributed to emotional and psychological well-being and acceptance of their condition.

#### Setbacks.

Exertion-induced symptom exacerbations, or setbacks, were frequently described. The exacerbations could range from temporary worsening of individual symptoms to prolonged reductions in physical function lasting days or weeks. These episodes negatively affected both physical capacity and psychological well-being, often leading to frustration, anxiety, and reduced motivation. While the temporary setbacks and symptom flare-ups in connection with the exercise were generally perceived as manageable and non-threatening by the participants, the recurrent and prolonged setbacks were more severe as they undermined the participants’ self-image, diminished their confidence in exercise as a strategy of recovery, and negatively affected their mental well-being.

*“It was really tough doing those exercises, because I always got worse afterwards and developed other problems instead, like new symptoms flaring up. Then you start thinking, ‘How much should I expose myself to? How much can my body handle? Can I cope with more symptoms?’”*

*-Participant E*

Although participants linked setbacks to exertion, the deteriorations were not solely related to exercise. Setbacks were described as unpredictable, with participants noting that what worked one day might fail the next. In this context, the individually tailored adaptations, such as modification of exercises, flexible intensity, and flexible modalities to carry out the intervention, were perceived as beneficial by the participants.

#### Daily life.

The reported improvements in physical, mental and cognitive functioning are described by participants as being connected to improved capability to engage in previously unmanageable activities – such as taking walks, attending family gatherings, or returning to work. Everyday tasks like shopping, cleaning, and reading also became possible or less challenging for some.

*“Yes, a huge difference. When I started, I couldn’t even play with the kids, barely at all, and now I can be in the garden and kind of play football… at least… not just standing and passing the ball, but I can run a bit and so on…”*

*-Participant F*

Despite these gains and meaningful improvements, many participants continued to experience substantial limitations where basic daily tasks were still very exhausting. Some described prioritising exercise over rest or other needs, which occasionally led to negative effects and reduced participation in other aspects of daily life.

#### Prerequisites and circumstances

The challenges of managing limited energy and the importance of rest, pacing, and tailored support are described in this category, together with findings of the role of support systems and multidisciplinary care, as all were considered important prerequisites to conduct the exercise intervention.

#### Energy distribution – A balancing act.

A central aspect of the participants’ experiences of the intervention concerns the management and distribution of limited energy resources. Energy was described as finite and required careful allocation across physical, cognitive, and social activities. Rest and recovery were perceived as fundamental needs; however, the amount varied significantly between individuals, from brief pauses to several days of recovery.

*“But it’s kind of like I try, for example, I don’t plan to both shower and do some other… now I don’t do very strenuous things. But for me it could be emptying the dishwasher, showering, changing the sheets in the bed – I don’t do that on the same day because it rarely turns out well. You have to plan your activities.”*

*-Participant G*

Participants emphasised the necessity of incorporating rest and energy management into future rehabilitation and training programs. Activity pacing, prioritisation, and self-monitoring were perceived as effective energy management strategies.

#### Support.

Support was expressed to be a central factor in the rehabilitation process, highlighting the input of support from healthcare providers, government agencies, workplaces, and personal networks. The presence or absence of support across these domains significantly influenced participants’ capacity to manage both rehabilitation and daily life.

##### Informal support.

Support and understanding from the surrounding environment, including family, friends, and collegues, were identified as critical enablers of recovery and rehabilitation. A clear discrepancy was noted between informal and formal support. Family, friends and peer support were frequently described as helpful and valuable, such as offering practical assistance and access to informal medical knowledge. Participants with limited social resources (such as being a single parent, living far from friends/family, or having children with special needs) reported specific challenges in balancing daily responsibilities with rehabilitation. To have informal support was associated with improved well-being and greater capacity to engage in rehabilitation, while its absence often led to increased stress, strain, and, in some cases, deteriorating health.

##### Formal support.

Formal support from welfare institutions and employers was more often perceived as insufficient, where participants described situations of premature termination of sick leave or pressure to return to work, and was linked to reduced energy management and financial strain. In contrast, those who experienced adequate formal support described having flexible workplaces and less financial stress, which facilitated their recovery process.

##### Multidisciplinary support.

It emerged that multidisciplinary support was important to the participants. In addition to physiotherapists, participants frequently highlight the need for occupational therapists, psychologists, dietitians, social workers, psychotherapists, and various medical specialists. Experiences of interdisciplinary collaboration vary widely among participants, ranging from highly coordinated care to fragmented or conflicting interventions. Regardless of individual experiences during the intervention period, there is a shared opinion and consensus to integrate additional professionals, particularly occupational and psychological support, into PCC-POTS rehabilitation frameworks.

*“Well, the way it has been for me, it hasn’t just been a physiotherapist who could help me with the rehabilitation, so much more has been needed. An occupational therapist has been needed, a dietitian has been needed, and really, so many people in the rehab team have been needed, and then the entire medical staff. And for me, also a psychologist, whom I chose to contact quite early because… well, just this thing with coping strategies, I think we tend to lose sight of that quite easily in rehabilitation.”*

*-Participant H*

Having other diagnoses or comorbid conditions (arthritis, Ehlers-Danlos syndrome, fibromyalgia, mast cell activation syndrome, re-infection in COVID-19, etc.) were barriers to adherence and adaptation. Recurrent infections were specifically identified as a common barrier to exercise and caused prolonged breaks from rehabilitation. Cognitive impairments, including brain fog and mental fatigue, further complicated participation. Participants emphasised that optimised pharmacological treatment prior to exercise initiation was essential, as inadequate medication reduced the efficacy or prevented participation. Appropriate medical management, such as cardiovascular agents and antidepressants, was identified to enhance both well-being and exercise capacity. Non-pharmacological strategies, including lifestyle changes and diets, reducing sensory input, using compression garments and assistive devices, were also perceived as beneficial.

##### Balancing hope and expectations

This main category comprises two generic categories: “Motivation and Expectations”, “Commitment, Emotions and Coping Strategies” to describe how goals, motivation, and emotional challenges shaped the participants’ experience of the rehabilitation intervention.

#### Motivation and expectations.

A prominent motivator that was described was the hope of regaining their pre-illness lives. The opportunity to take part in an exercise intervention gave the participants hope, motivation and optimism for the future. The structure of the intervention served as a motivational tool and a tool for planning and goal setting, encouraging participants to strive toward higher levels of physical activity.

*“On a spiritual level, incredibly hopeful, and then cognitively or behaviorally, it was really nice to get a clearly outlined program, so I was probably very motivated. I was very hopeful.”*

*-Participant I*

Participants highlighted the value of setting both long-term and short-term goals to monitor progress and maintain motivation. Due to the unpredictable nature of post-COVID recovery, goals often required adjustment during the rehabilitation, and the participant described the adjustment as a careful balance between hope and realism.

Initial optimism and expectations about the intervention, often seen as a comprehensive solution, were later tempered by the realisation of slower or limited progress than expected. Despite this, most participants maintained a belief in the benefits of exercise. However, sustaining motivation proved challenging to the participants when improvements were lacking. For some, the absence of large health gains led to disappointment, reduced motivation and lower adherence.

#### Commitment, emotions and coping skills.

The participants’ expectations and strong beliefs in exercise treatment contributed to a dedicated commitment and adherence to the intervention. Participants viewed it not merely as a treatment modality, but as their primary pathway to improved health, and thereby engaged in the exercise treatment without considerations.

Previous effective coping strategies, which commonly involved strenuous physical activity, were described as no longer reliable or accessible due to their illness and exercise intolerance. The loss of familiar coping strategies contributed to a perception of losing control and difficulties in managing their new situation.

*“… I’ll just go for it, and then it doesn’t work, and you think now I’ve decided, like… no fuss, let’s go… and all those things that used to work, they don’t work now. It becomes extra clear that this is how it is now, this is how things are, and that’s what’s hard – how it turns out when you don’t know who you’ll be today…”*

*-Participant D*

The perception of losing control was tied to feelings of shifted identity and existential concerns. In addition to this, a broad spectrum of other emotions was present, such as experiencing frustration, self-criticism, and disappointment with their own perceived performance. Some participants also experienced fear related to exercise – fear of performing or fear of avoiding it, or fear of causing exertional malaise (PEM). The fear was often related to previous experiences prior to the feasibility study, where exercise had caused symptom exacerbation, and led to caution in daily activities for some of the participants.

Despite emotional and psychosocial challenges, participants described a gradual process of psychological adaptation and growing acceptance and confidence in their medical condition and abilities. They describe developing strategies to manage setbacks as part of the rehabilitation, which were mediated by the intervention structure, the professional support and time. The coping strategies included mental and pacing preparation, self-reflection, flexible exercise routines, and a gradual shift in mindset regarding physical activity. The strategies were considered as a key factor in rebuilding hope, confidence and self-compassion and thereby contributed to long-term adherence to exercise and well-being.

*“Right now, it’s kind of like I’m trying to relearn, to try to listen more to my body and to try to think that I should become better than I am today, and not that I should become healthy…. // … I might not be able to do the same things I did before, and maybe I shouldn’t have done those things before either, but it’s just that I’m used to gritting my teeth and pushing through, you know. So, this is the first time in my life that I’m kind of listening to my body.”*

*-Participant C*

## Discussion

This study is, to our knowledge, the first to explore patients’ experiences of exercise interventions in the PCC-POTS population. Key insights include the importance of individual tailoring and continuous adaptation of exercise programs to ensure feasibility, and the need for adequate support – such as social and behavioural support, and access to multidisciplinary and medical care – to enable participation and to benefit from physical exercise intervention.

The category “Content and Structure of the Intervention” revealed that individual tailoring was essential for participants’ engagement in exercise. The importance may reflect the marked heterogeneity in symptoms and exercise tolerance observed in PCC and POTS [11,25,28,32,53] and likely contributed to the varying needs and preferences [35,36], Although dimensions of progression and dosage are mentioned by Estebanez-Pérez et al. [44] and Kok et al. [40], the experiential aspects of exercise content remain largely unexplored and rarely address the need for individualisation in PCC. Our findings align with Walsh et al (2025) [48], indicating that experiences of resistance versus aerobic exercises, as well as preferences for structured or flexible regimens, differ substantially between individuals. This underscores that the modality and content of exercise interventions are not self-evident. Individualisation is recommended to ensure feasibility in both clinical practice and research contexts [32,37–39], and our findings nuance and extend this by providing practical implications for intervention content and adaptation. The positive experiences of group sessions is consistent with previous studies [41,45–47], and may reflect the value of social validation and shared illness experiences, and online formats may serve as a valuable alternative, as digital solutions are generally well-received in PCC rehabilitation [45,47].

The importance of close monitoring and validation by a physiotherapist aligns with previous research [41,44–48], highlighting trust, consistent practitioner support and a strong therapeutic alliance as key facilitators of engagement [54] The need for such professional support may reflect the uncertainty associated with PCC-POTS, including fluctuating symptoms, symptom exacerbations, an unpredictable prognosis, limited treatment options, and, for many individuals, a challenge of adapting to a sudden disruption of daily life. Together these factors may increase the need for professional reassurance and individualized guidance regarding exercise. The need for security, continuity of care, and opportunities to have questions answered and individual needs addressed may, depending on the structure of the healthcare system, also be fulfilled by other healthcare professionals, such as nurse navigators or through structured interdisciplinary teamwork [55,56]. While the importance of healthcare professionals’ knowledge has been recognized within PCC [41,57], it is more prominent in POTS research, where patients express a desire for greater awareness and expertise among healthcare providers [48,58,59]. Our findings, together with previous research in digital PCC rehabilitation [40,41,44,45,47], suggest that educational materials are a valuable component of rehabilitation, as they may support both healthcare professionals and patients, improve understanding of symptoms and strategies, and enhance the delivery of rehabilitation and care,

A notable finding was that some participants experienced a sense of dependency on their physiotherapist, which warrants consideration, as it may reduce openness and limit necessary adjustments of the intervention [60]. While a strong therapeutic alliance appeared important for engagement, an overreliance on professional guidance may have hindered the development of self-management skills and ability to independently adapt exercise [54]. The dependency described by some participants may also reflect the strong desire to achieve progress in rehabilitation and return to higher levels of activity, as well as perceived expectations to demonstrate improvement to the physiotherapist. Such desires or expectations may have influenced participants’ willingness to openly communicate the challenges or setbacks [60]. These findings highlight the importance of balancing professional support with strategies that promote autonomy and self-efficacy. The findings also have implications for the design and content of future interventions, suggesting that professional support should be balanced with gradual transition towards greater self-management and sustainable engagement in physical activity [54,60].

Findings related to outcomes and effects, presented in the category “Impact of the tailored exercise” are largely aligned with previous research on COVID-19, PCC, and POTS [40,44,45,47,48,57,61,62],. The similarities in experiences suggest that non-linear recovery and fluctuating symptoms represent a core characteristic of the conditions, potentially influencing outcomes regardless of intervention type. As reported in previous studies [45,61], fatigue was particularly burdensome and may indicate that, although exercise may improve physical or mental health, improvements do not necessarily translate into recovery or restored functioning in daily life. Given the broad range of outcomes experienced by participants, future studies may incorporate a wide range of objective assessments, including measures of physical, psychological, cognitive and exertion-related outcomes, to better understand the overall effects of exercise interventions in PCC-POTS..

The category “Prerequisites and Circumstances” revealed the importance of social context and energy management, suggesting that successful participation in exercise is influenced not only by the intervention itself but also by the behavioural, structural and environmental surroundings. Although not formally included in the feasibility trial, many participants learned or adapted pacing strategies. Variation in pacing strategies may reflect differences in the physiotherapists’ expertise, the interdisciplinary support, or participants’ readiness for behavioural change [63,64]. The need for energy management is consistent with previous research [46,48,53,58,65–68], and our results support that pacing may be considered a core component of PCC or POTS rehabilitation. Integrating energy management may reduce PEM, prevent setbacks, and support sustainable physical activity [53,65,68].

Although participants’ attitudes and engagement influenced all categories, their experiences emerged as a distinct category of their own: “Balancing Hope and Expectations”. Similarities with findings from recent studies of COVID-19 [62] and POTS [58] suggest that disorientation, fear, and frustration are common psychological challenges across the conditions. In line with Walsh et al. (2025) [58], participants described processes of acceptance and the development of coping strategies to establish a new sense of normality. This also aligns with the biopsychosocial approach in physiotherapy and long-term conditions [69–72] as well as the use of behavioural interventions such as Acceptance and Commitment Therapy (ACT) in PCC [31].

While it remains unclear whether behavioural change techniques were explicitly applied, the exercise program itself may have functioned as exposure therapy, potentially reducing fear and promoting behavioural adaptation [73]. A striking finding was the high level of commitment demonstrated by participants, which may reflect a strong internal drive for recovery and a broader belief in the value of physical exercise. This has been described previously by Walsh et al. (2025) in patients with POTS, alongside the role of motivation and self-efficacy. The ability – or, in some cases, the inability – to adjust pacing and exercise routines may reflect participants’ individual level of psychological flexibility and behavioural awareness [63,64]. Greater psychological flexibility may mediate participants’ adaptation and enhance the benefit from the intervention, as seen in ACT-based treatments [74]. This level of commitment has not been described in previous intervention studies for PCC, but high motivation to participate in research has been noted [75,76].

Taken together, these findings suggest that future rehabilitation interventions for individuals with PCC-POTS may benefit from prioritizing individualized exercise prescription and may include both endurance and resistance exercises. Such exercise interventions may be complemented by education on pacing and energy management, as well as by additional physiotherapy, psychological or social interventions when needed. Based on the participants’ experiences, regular reassessments and adaptation of exercise content and dosage to individual needs appeared important. The positive experiences of regular access to professional guidance, opportunities for peer support, and interdisciplinary collaboration suggest that these components may be valuable to include in rehabilitation interventions, as they may further enhance engagement and overall experiences, and could potentially contribute to improved health outcomes in this population.

### Methodological considerations

A strength of the study is the use of individual interviews, which likely enabled flexible adaptation to individuals’ needs. The interviewers’ independence from both the participants and the feasibility study likely reduced power imbalances and social desirability bias, thereby encouraging open and honest responses while enabling a more neutral approach to the interviews with fewer preconceptions during data collection. The interviewers’ different professional and personal backgrounds may have influenced the content of the interviews but also strengthened the study by capturing a broader range of perspectives among participants. As both interviewers were experienced in research settings, they had skills to obtain meaningful data. However, more structured interviewer training or calibration during the data collection phase might have yielded even more in-depth information on certain aspects of the participants’ experiences. Although field notes or structured member checking were not employed, interviewers continuously clarified responses during interviews to support face validity through accurate understanding and interpretation [77]. The credibility and confirmability might have been strengthened if participants validated the findings, but this was consciously left out since it is not an established verification strategy [77,78].

The criterion-based sampling was appropriate according to the research aim, and the sample represented the population with variation in age, gender, life situation, and level of disability. Category replication was evident, as later interviews primarily contributed additional examples and nuances within existing codes and categories. No new codes, categories or substantial insights emerges, suggesting that the data had achieved sufficient richness and captured the most relevant perspectives. However, earlier preliminary analysis might have identified saturation sooner [78].

During analysis, the codebook and manifest coding supported credibility, dependability, and investigator triangulation by fostering shared understanding among authors [77–79]. As most of the researchers was involved in the feasibility trial, prior knowledge of the intervention, population and the clinical challenges existed before the analysis commenced. While this facilitated an in-depth understanding of participants’ accounts, it also entailed a risk of preconceived assumptions. Reflexivity was therefore maintained throughout the study through repeated reflection on emerging interpretations, and discussions within the research team aimed at challenging pre-understandings and enhancing analytical rigor. The researchers’ different backgrounds and varying levels of involvement in the feasibility trial enriched the analytical discussions and facilitated consideration of alternative interpretations. Regular discussions within the research team, together with repeated comparisons with the original transcripts, helped ensure that the findings remained grounded in participants’ experiences.

A limitation is that all participants contracted COVID-19 in 2020, which may reduce the transferability to later cohorts given the potential evolution of healthcare practices, awareness, diagnostic pathways, clinical management and rehabilitation services [2,3,15,28,43]. Consequently, the experiences of individuals infected later in the pandemic may differ from those represented in this study. The extent to which the findings also apply to individuals with POTS of other etiologies also remains uncertain. However, participant characteristics were consistent with those reported in other PCC and POTS populations [4,7,10,13,16,17,28], suggesting partial transferability. At the same time, individuals with PCC-POTS can experience a broader symptom burden ([7,13,15,28], which may influence both exercise adaptations as well as the experiences of exercise. The findings should also be interpreted with caution in populations with different sociodemographic characteristics, other levels of health literacy, healthcare contexts, or exercise interventions.

## Conclusion

This study underscores the complexity and individual variability in experiences of individually tailored exercise interventions for individuals with PCC-POTS. The findings highlight the necessity of a flexible rehabilitation approach that accommodates symptom fluctuations, supports pacing and energy management, and responds to diverse psychosocial needs and resources. Support from a knowledgeable physiotherapist proved central to participants’ engagement, with trust, continuity, and clinical expertise in PCC and POTS serving as key facilitators of adherence and perceived safety. Integrating individually tailored physical exercise with gradual progression, alongside psychological and social support, ultimately influenced a more successful recovery and enhanced long-term well-being. Overall, the findings contribute to a growing body of knowledge that may inform future therapies, strategies, and patient engagement, suggesting that rehabilitation for PCC-POTS should be holistic, multidisciplinary, and tailored. Future trials should investigate the effectiveness of individualized exercise, pacing, and multidisciplinary support for PCC-POTS.

## Supporting information

S1 Appendix Interview Guide for Rehabilitation after COVID-19, POTS. (DOCX)

## Abbreviations

ACT: Acceptance and Commitment Therapy
ADL: Activities of Daily Living
PCC: Post COVID-19 Condition
PEM: Post Exertional Malaise
POTS: Postural Orthostatic Tachycardia Syndrome.
