# Supplementary material for: Experiences of individually tailored physical exercise in COVID-19-induced postural orthostatic tachycardia syndrome: A qualitative content analysis
Source: PLoS One. 2026 Nov 4;21(11):e0360748. doi: 10.1371/journal.pone.0360748 (PMC13734440; doi:10.1371/journal.pone.0360748)
Supplement: Supplementary file 1 [file pone.0360748.s001.docx]

## Interview Guide for Rehabilitation after COVID-19, POTS

**Information Beforehand -** Introduction. The purpose of the study, confidentiality, and the right to withdraw participation at any time. When it comes to experiences, there are no right or wrong answers.

**Purpose** – to explore the person’s experiences of individually tailored physical exercise for POTS after COVID-19.

**Suggested Introductory Questions**

- What does rehabilitation mean to you?
  - Do you have any previous experience in rehabilitation of any kind?
- How would you describe your symptoms of POTS after COVID-19? Any persistent symptoms?

**Suggested Intervention-specific questions**

- How did you perceive the rehabilitation/treatment?
- What are your thoughts about the structure of the training you performed? (*E.g. How long/how often/how strenuous?)*
- Do you feel that your symptoms (POTS) were affected/changed when you performed the training or immediately after? Can you describe how?
- Can you describe an occasion where you received good help from the treatment/training, or no help at all or had the opposite effect, e.g., worsened your symptoms?
- Did you ever consider quitting the treatment? What were your thoughts then?
- What expectations and/or personal goals did you have for the rehabilitation in the study? What has been the most significant aspect of rehabilitation in the study?
- What support did you receive, and/or the support you needed in rehabilitation?
- What have you been able to do yourself *(own strategies for persistent problems)*?
- Regarding the follow-up visits with the physiotherapist in the study. Could you describe the structure's appearance? How did you perceive the visits? What are your thoughts about it?
- Were other professionals involved in the rehabilitation, and what did the different professionals do?
