# Supplementary material for: Experiences of individually tailored physical exercise in COVID-19-induced postural orthostatic tachycardia syndrome: A qualitative content analysis
Source: PLoS One. 2026 Nov 4;21(11):e0360748. doi: 10.1371/journal.pone.0360748 (PMC13734440; doi:10.1371/journal.pone.0360748)
Supplement: Supplementary file 2 [file pone.0360748.s004.docx]

**Response to Reviewers**

We would like to thank the Editor and the Reviewers for their careful assessment of our manuscript and for the thoughtful and constructive comments provided. The feedback has helped us to further strengthen the manuscript, particularly regarding methodological transparency, interpretation of the findings, and discussion of their implications. We have carefully considered all comments and have addressed each point and revisions in detail below.

| **Journal Requirements** | | |
| --- | --- | --- |
| **1** | Please ensure that your manuscript meets PLOS ONE's style requirements, including those for file naming. | Thank you, we have revised the manuscript and associated files to ensure compliance with PLOS ONE requirements and according to the journal templates. |
| **2** | In the online submission form, you indicated that the data underlying this study consist of qualitative interview transcripts containing sensitive and identifiable personal information. Due to ethical and legal restrictions, including approval requirements from the Swedish Ethical Review Authority (ethical approval 2020-02149, including amendments) and participants' informed consent, the data cannot be made publicly available. Even in pseudonymised form, the data cannot be fully de-identified without risking participant confidentiality. Data may be made available upon reasonable request, subject to approval by the responsible research authority and in accordance with applicable data protection regulations, including the EU General Data Protection Regulation (GDPR). Access requests should be directed to the corresponding author, the primary investigator or the Karolinska Institutet Research Data Office (rdo@ki.se), and will be evaluated on a case-by-case basis under conditions that ensure the protection of participant privacy.  All PLOS journals now require all data underlying the findings described in their manuscript to be freely available to other researchers, either 1. In a public repository, 2. Within the manuscript itself, or 3. Uploaded as supplementary information. This policy applies to all data except where public deposition would breach compliance with the protocol approved by your research ethics board. If your data cannot be made publicly available for ethical or legal reasons (e.g., public availability would compromise patient privacy), please explain your reasons on resubmission and your exemption request will be escalated for approval. | We understand and appreciate the rationale behind your data-sharing policy. However, due to ethical and confidentiality considerations, we are unfortunately unable to make the data publicly available. The data underlying this study consists of qualitative interview transcripts containing sensitive personal information and public sharing is not permitted under the study's ethical approval or the participants' informed consent. We appreciate your understanding of these limitations and are happy to provide additional details upon request. |
| **3** | We note that the grant information you provided in the ‘Funding Information’ and ‘Financial Disclosure’ sections do not match.  When you resubmit, please ensure that you provide the correct grant numbers for the awards you received for your study in the ‘Funding Information’ section. | Thank you for noticing this. We have reviewed and revised the funding information to ensure consistency between the Funding Information and Financial Disclosure sections. |
| **4** | [Please include captions for your Supporting Information files at the end of your manuscript, and update any in-text citations to match accordingly. Please see our Supporting Information guidelines for more information: http://journals.plos.org/plosone/s/supporting-information.](https://eur01.safelinks.protection.outlook.com/?url=http%3A%2F%2Ftrack.editorialmanager.com%2FCL0%2Fhttp%3A%252F%252Fjournals.plos.org%252Fplosone%252Fs%252Fsupporting-information.%2F1%2F010f019f97a83567-63e2914d-4f7f-402b-8760-dbf7266853ab-000000%2F5d7yXnFgv583bsDZuUG7P1wLxHElvt778_R2uNOzETY%3D258&data=05%7C02%7Cannie.svensson%40ki.se%7C94de80cf69354b6c18e908deea0a1347%7Cbff7eef1cf4b4f32be3da1dda043c05d%7C0%7C0%7C639205526473942717%7CUnknown%7CTWFpbGZsb3d8eyJFbXB0eU1hcGkiOnRydWUsIlYiOiIwLjAuMDAwMCIsIlAiOiJXaW4zMiIsIkFOIjoiTWFpbCIsIldUIjoyfQ%3D%3D%7C0%7C%7C%7C&sdata=UzfTbPH8HR%2FpMAv3Yr0ham3Pi5IiHwrbR2tlDsY7MXc%3D&reserved=0) | Thank you, we have now added a Supporting Information section at the end of the manuscript, including a caption describing the supporting information file. |
| **5** | If the reviewer comments include a recommendation to cite specific previously published works, please review and evaluate these publications to determine whether they are relevant and should be cited. There is no requirement to cite these works unless the editor has indicated otherwise. | Thank you for this note. We reviewed all comments and confirmed that no specific recommendations for additional citations were made. Consequently, no changes to the reference list were necessary in relation to this point. |
| **Reviewer 1** | |  |
| **1** | Researcher reflexivity Although the authors describe the professional backgrounds of the research team and acknowledge their previous involvement in the feasibility study, a more comprehensive discussion of researcher reflexivity would improve methodological transparency. Specifically, the manuscript would benefit from explaining how potential preconceptions were managed throughout data collection, coding, and interpretation. | Thank you for raising this important comment. We agree that the description of researcher reflexivity required further elaboration. We have therefore expanded the Methodological Considerations section to provide a more detailed account of how potential preconceptions and prior involvement were managed throughout the study.  *Page 29-30* |
| **2** | Data sufficiency The manuscript states that category replication was achieved; however, the process used to determine that sufficient information had been collected is not fully described. Providing additional details regarding how data adequacy or conceptual saturation was evaluated would strengthen the credibility of the qualitative findings. | We appreciate the opportunity to clarify this point and we have now revised the methodological considerations to clarify how category replication was assessed during the analytic process, supporting that sufficient information had been collected to address the study aim.  *Page 29* |
| **3** | Sample characteristics and transferability Most participants were female, highly educated, and recruited from a single specialized rehabilitation program. Although acknowledged as a limitation, a more detailed discussion regarding how these characteristics may influence the transferability of the findings to other healthcare settings and patient populations would be valuable. | Thank you for this valuable comment. We have expanded the methodological considerations section to provide a more detailed discussion of transferability. Specifically, we now discuss how the characteristics of the participants and the setting for the intervention may influence transferability to other populations and healthcare settings.  *Page 30* |
| **4** | Influence of participation in the feasibility trial All participants had previously completed the feasibility intervention. This context may have influenced their perceptions through expectation effects, gratitude bias, or positive engagement with the research team. Expanding the discussion of these potential influences would enhance the interpretation of the findings. | Thank you for this very important comment. We agree that participation in the feasibility trial may have influenced how participants perceived and described their experiences. We have expanded the Methodological Considerations section of reflexivity and limitations. Furthermore, we expanded the Discussion to further discuss how participants’ strong motivation for recovery, perceived expectations of progress, and dependency on professional support may have influenced the intervention and its adaptation. We believe these revisions provide a more nuanced interpretation of the findings.  *Pages 25-26, 29-30* |
| **5** | Integration of findings with previous literature The discussion is comprehensive but occasionally reiterates the results before critically interpreting them. Greater emphasis on explaining similarities and differences with previous qualitative studies, as well as discussing possible underlying mechanisms, would further enrich the manuscript. | We are grateful for this constructive feedback, and the comment helped us improve the manuscript. We have revised the Discussion to reduce repetition of the results and place greater emphasis on critical interpretation of the findings and of potential mechanisms. We believe these revisions strengthen the interpretation of the findings and improve the integration with the existing literature.  The revisions are incorporated throughout the Discussion section *(pages 23-28).* |
| **6** | Clinical implications The manuscript appropriately concludes that individualized exercise and expert physiotherapy supervision are important. However, the discussion could be strengthened by providing more practical recommendations regarding which intervention components should be prioritized in future rehabilitation programs. | Thank you for this helpful suggestion. In response, we have added a paragraph at the end of the Discussion section outlining the clinical implications of our findings while also incorporated throughout the Discussion.  *Page 28* |
| **7** | Patient dependency on physiotherapists One particularly interesting finding is that some participants developed a sense of dependency on their physiotherapist. This deserves further exploration, particularly regarding strategies that may promote patient autonomy and self-management while maintaining adequate professional support. | We appreciate this thoughtful suggestion, and we agree that the reported sense of dependency is an interesting finding that deserves further emphasis. We have revised and expanded the Discussion to further explore the potential mechanisms and implications of this finding *(pages 25-26).* Furthermore, we have incorporated an implication for future studies, emphasizing the importance of balancing professional support with transition and strategies to develop skills and confidence in long-term self-management. These additions can be found in the Discussion section *(pages 25-26).* |
| **8** | Presentation of qualitative findings The participant quotations effectively support the identified categories. However, a few sections contain multiple quotations conveying similar ideas. Reducing some repetitive quotations may improve readability without compromising methodological rigor. | Thank you for this helpful comment. We have revised the manuscript and removed a quotation from the Result Section.  *Page 22* |
| **9** | Conceptual model Figure 1 adequately summarizes the thematic categories. Nevertheless, an additional conceptual figure illustrating the relationships among the five main categories could enhance readers' understanding of the proposed rehabilitation framework. | Thank you for this valuable suggestion, and a conceptual model illustrating relationships may enhance understanding. However, the aim of the study was to describe participants' experiences rather than to develop a theoretical or conceptual framework. We therefore chose to retain Figure 1 in its current form, which we believe adequately summarizes the findings while remaining close to the data. |
| **10** | Future research The findings identify several potentially important components of rehabilitation, including individualized exercise prescription, pacing strategies, psychological support, patient education, and multidisciplinary care. Highlighting these elements as priorities for future randomized controlled trials would strengthen the conclusion. | We appreciate that the reviewer raises this important point, we have now revised the Discussion and Conclusion sections to highlight priorities for future research to assess effectiveness of these elements of rehabilitation.  *(Page 26, 28, 31)* |
| **Reviewer 2** | | |
| **1** | How was the sample size calculated and how was the decision to take the study population from the other study taken? | Thank you for this comment. As this was a qualitative interview study, no formal sample size calculation was performed, as such calculations are generally not applicable in qualitative research.  As the aim of this study was to explore experiences of a specific exercise intervention, it was considered necessary to recruit individuals who had participated in the intervention, and there for the described purposeful selection was used.  The data adequacy was then evaluated during the analytical process where category replication was evident. We have further clarified this data evaluation in the revised Methodological Considerations section.  *Page 29* |
| **2** | Line 109- Why was the criterion based sample method used here? Give reasoning. | Thank you for highlighting this methodological consideration. Please also see our response to Comment 1. The inclusion criteria for this study were participation in the feasibility trial and experience of exercising according to the intervention, regardless of the extent to which the intervention had been completed or delivered. We considered these criteria essential to ensure that participants could provide relevant experiences regarding the intervention. We have now clarified the recruitment in the Methods section.  *Page 5* |
| **3** | What was the significance of noting and mentioning the marital status of the study population? | We appreciate this observation regarding the participants. Marital and parental status, employment and sick leave were included as descriptive characteristics to provide a broader description of the participants’ life circumstances. Family-related and social contextual factors may influence the practical conditions for managing both everyday life and participation in rehabilitation programs. Therefore, we considered it relevant to report the participants marital and parental status as part of the context and diversity of the sample.  We have elaborated on the transferability of the findings in the Methodological Considerations section, *page 30* |
| **4** | Significance of Partial and complete sick leaves taken. | Thank you for this comment. Please see our response to Comment 3. |
| **5** | Line 127- Were the interviewers trained to take the interviews by the researcher? | Thank you for this valuable comment on design and methodology. Both interviewers had previous experience conducting interviews in research settings, and therefore no specific interviewer training was conducted prior to the study. However, we agree that additional interviewer calibration or structured training might have further enhanced consistency and depth across interviews and we have clarified this in the Methodological Considerations section *(page 29).* |
| **6** | Line 127- Significance of Interviewer’s personal experience with PCC? Bias? | Thank you for highlighting this issue. One of the interviewer's personal experiences with PCC may have influenced the interviews. To address this, we have expanded the discussion of reflexivity in the Methodological Considerations section *(page 29).* |
| **7** | Line 190- What was done for resistance training? Not specified. | Thank you for this comment. All components of the intervention were individually tailored and therefore varied between participants according to their symptoms, functional capacity, and progression throughout the intervention. Detailed information regarding the intervention has been published previously in the feasibility trial (<https://doi.org/10.1038/s41598-024-71055-5>), while the aim of the present qualitative study was to explore participants’ experiences of the intervention. For this reason, we have chosen to refer readers to the feasibility study for a comprehensive description of the intervention and have not expanded this section further in the current manuscript. |
| **8** | Line 227- What kind of low intensity exercises were given? not mentioned in the study. | Thank you, please see our response to Comment 7. |
| **9** | Line 239- Group based training? Most of the study mentions customization. | Thank you for this comment, please also see our response to Comment 7.  The term "group-based training" refers to a shared exercise setting rather than a standardized group exercise program. Some participants had the opportunity to exercise in this setting, where they performed their tailored exercise program alongside other individuals with PCC-POTS. More detailed information regarding the content of the intervention has been published in the feasibility trial (<https://doi.org/10.1038/s41598-024-71055-5>) |
| **10** | Line 245- How was supervision done during the home based program? How did the researcher make sure that the study population exercised at home and that there was no need to increase the intensity of exercises? | Thank you for this comment. The procedures regarding supervision, adherence, and progression of the exercise are described in detail in the previously published feasibility trial (<https://doi.org/10.1038/s41598-024-71055-5>). As the primary aim of the present study was to explore the participants’ experiences of the intervention, we chose not to provide further details to this manuscript. |
| **11** | Line 247- Reasoning for addition of cognitive tasks in the Physiotherapy Treatment. Also how was standardization of Physiotherapy treatment done? | Thank you for this comment. The cognitive tasks and relaxation exercises in the Result Section were derived from some participants’ descriptions of their rehabilitation experiences. These were described by participants as complementary strategies or treatments that they found helpful in managing their condition.  Regarding the standardization of physiotherapy treatment, the intervention and its procedures have been described in the previously published feasibility trial (<https://doi.org/10.1038/s41598-024-71055-5>). To improve clarity, we have revised the manuscript to clarify that cognitive and relaxation exercises originated from participants’ accounts of their rehabilitation experiences.  *Page 12* |
| **12** | Line 257- Reasoning for promotion of relaxation. | Thank you, please see our response to comment 11. |
| **13** | Line 259- Trust and validation. Why was this studied? | Thank you, we are grateful to clarify this point. Trust and validation were not predefined areas of interest prior to data collection. Rather, these concepts emerged from the analysis of interview data, where participants described these aspects as important components of their interaction with the physiotherapist and their experiences of the intervention. We therefore considered these findings important to report as part of the results. No changes to the manuscript have been made in response to this comment. |
| **14** | Line 265- Psychological well being was not described as an objective. | Thank you for this comment, as with trust and validation, psychological well-being was not a predefined objective or outcome of the study. But it emerged inductively from participants during the interviews as a meaningful aspect of their experiences and outcomes of the intervention. To stay with the exploratory nature of the study, we considered it important to report this as part of the findings. Therefore, no changes to the manuscript have been made in response to this comment. |
| **15** | Line 267- Continuity of care. How was this studied? | Thank you, se also our response to comment 10. Continuity of care was not explicitly studied in this qualitative study, but participants described this as a factor that influenced the experiences of the intervention and their participation. We therefore considered it an important finding to report.  Information regarding the structure and continuity within the intervention is described in the previously published feasibility trial (<https://doi.org/10.1038/s41598-024-71055-5>). Consequently, no changes to the manuscript have been made in response to this comment. |
| **16** | Line 283- How was uniformity in treatment maintained? | Thank you, se also our response to comments 6, 7, 8, 10 and 11.  Participants’ perceptions and experiences of physiotherapists’ expertise are reported rather than an objective assessment of treatment uniformity. The participants described how they experienced the physiotherapists’ knowledge and understanding of PCC-POTS, and how this influenced their sense of safety and confidence in the intervention. As these findings reflect participants’ experiences, no additional assessment of treatment uniformity was undertaken within this qualitative study. Information regarding the intervention procedures and treatment delivery is available in the previously published feasibility trial (<https://doi.org/10.1038/s41598-024-71055-5>). |
| **17** | Line 319- Were patients with cognitive impairment included? | Thank you for observing this important aspect of participant diversity. Cognitive impairment was not an exclusion criterion for participation in this interview study, but both POTS and PCC may be associated with cognitive symptoms or reduced cognitive functioning. As the participants were recruited from the feasibility trial, the interview cohort shared the same overall profile of comorbidities and PCC-related symptoms (including cognitive symptoms), as reported in the previously published feasibility study (<https://doi.org/10.1038/s41598-024-71055-5>), where none of the participants had a documented diagnosis of cognitive impairment. As cognitive function was not systematically assessed, we chose not to further classify or describe participants according to cognitive status. However, our findings indicate that at least some participants perceived their cognitive functioning to be negatively affected by POTS or PCC *(Result Section page 15-17*). We therefore believe that additional reporting regarding cognitive function would not provide reliable information within the scope of the current study, and thereby, no changes to the manuscript have been made in response to this comment. |
| **18** | Line 330- How was cognitive improvement recorded and evaluated by the researcher? | We appreciate this question and comment. Cognitive functions, mental well-being, and fear of symptom exacerbation/PEM were not objectively recorded or evaluated as outcomes in the present qualitative study. These aspects were identified from the participants’ descriptions of their experiences and perceptions during the interviews and were therefore reported as part of the findings. As the aim of this study was to explore participants’ experiences of the intervention rather than to evaluate clinical outcomes, all three aspects were included as they were described as meaningful to their rehabilitation experiences.  Objective measures of emotional well-being, anxiety, and depression were collected as part of the feasibility trial using the PHQ-9 and GAD-7 questionnaires and are reported in the previously published feasibility study (<https://doi.org/10.1038/s41598-024-71055-5>). In contrast, cognitive function and post-exertional malaise (PEM) were not objectively evaluated in the feasibility trial. Given the findings, these aspects may be important to investigate in future studies of exercise interventions for individuals with PCC-POTS, which we now have clarified and added to the discussion.  *Page 26 - 27* |
| **19** | Line 334- How was emotional and Psychological well being monitored? | Thank you, please see our response to comment 18 |
| **20** | Line 495- How was fear of causing external malaise evaluated? | Thank you, please see our response to comment 18 |
| **Reviewer 3** | | |
|  | Yes, the manuscript is technically sound and the conclusions are supported by the data presented. The sample size has not been calculated, but the reasons for choosing the particular sample size has been mentioned. The sample shows a preponderance of female subjects which should be mentioned in the conclusion. | Thank you for highlighting this preponderance of females. We acknowledge that the dominance of female participants may influence the transferability of the findings, although this sex distribution also reflects the clinical demographics of the condition. We chose to further elaborate on the transferability of the findings in the Methodological Considerations section. We believe that this provides a more appropriate context for interpreting the transferability of the findings than the conclusion section.  *Page 30* |
|  | The analysis has been performed appropriately and rigorously, as required for a Qualitative study. The software used for the analysis and the procedure has been explained in detail. | Thank you for your positive feedback, we appreciate the reviewer’s recognition of the analytical process and the clarity of the methodological description. |
|  | The authors have not made all data underlying their findings available, citing ethical reasons. However the authors have mentioned that it can be made available through proper channels in case of reasonable request | Thank you, we appreciate the reviewer’s recognition of the ethical considerations underlying our data-sharing statement. |
|  | The manuscript is presented in an intelligible manner and written in standard English. The language is clear, correct and unambiguous without any typographical and grammatical errors. | We appreciate your positive feedback on our manuscript. |
