# Supplementary material for: Experiences of individually tailored physical exercise in COVID-19-induced postural orthostatic tachycardia syndrome: A qualitative content analysis
Source: PLoS One. 2026 Nov 4;21(11):e0360748. doi: 10.1371/journal.pone.0360748 (PMC13734440; doi:10.1371/journal.pone.0360748)
Supplement: Supplementary file 3 [file pone.0360748.s002.doc]

1. How was the sample size calculated and how was the decision to take the study population from the other study taken?
2. Line 109- Why was the criterion based sample method used here? Give reasoning.
3. What was the significance of noting and mentioning the marital status of the study population?
4. Significance of Partial and complete sick leaves taken.
5. Line 127- Were the interviewers trained to take the interviews by the researcher?
6. Line 127- Significance of Interviewer’s personal experience with PCC ? Bias?
7. Line 190- What was done for resistance training? Not specified.
8. Line 227- What kind of low intensity exercises were given ? not mentioned in the study.
9. Line 239- Group based training?Most of the study mentions customization.
10. Line 245- How was supervision done during the home based program? How did the researcher make sure that the study population exercised at home and that there was no need to increase the intensity of exercises?
11. Line 247- Reasoning for addition of cognitive tasks in the Physiotherapy Treatment. Also how was standardization of Physiotherapy treatment done?
12. Line 257- Reasoning for promotion of relaxation.
13. Line 259- Trust and validation. Why was this studied?
14. Line 265- Psychological well being was not described as an objective.
15. Line 267- Continuity of care. How was this studied?
16. Line 283- How was uniformity in treatment maintained?
17. Line 319- Were patients with cognitive impairment included?
18. Line 330- How was cognitive improvement recorded and evaluated by the researcher?
19. Line 334- How was emotional and Psychological well being monitored?
20. Line 495- How was fear of causing external malaise evaluated?
